# An Uncommon Cause of Dysphagia: Postpneumonectomy Syndrome

**DOI:** 10.1155/2021/6658690

**Published:** 2021-03-08

**Authors:** Erica Rego, Ahmed Abdelmeguid, Yuqi (Kevin) Wang, Karuna Dewan

**Affiliations:** ^1^New Jersey Medical School, Rutgers University, New Brunswick, NJ, USA; ^2^Division of Laryngology, Department of Otolaryngology- Head and Neck Surgery, Stanford University, Stanford, CA, USA; ^3^Division of Neuroradiology, Stanford University, Stanford, CA, USA

## Abstract

**Objective:**

Dysphagia after pneumonectomy is uncommon but concerning. The purpose of this paper is to present a case of dysphonia secondary to postpneumonectomy syndrome. *Case Report*. A 66-year-old female with stage IIIa adenocarcinoma of the lung was treated with a left pneumonectomy. Three years later, she presented with severe dysphagia, dyspnea, and dysphonia. Esophagram demonstrated severely deviated esophagus to the left of midline, attributed to prior left-sided pneumonectomy, without clear evidence of any external compression. Chest CT scan showed associated leftward mediastinal shift. This patient was treated with voice therapy and an exclusion diet, as the patient elected not to have surgery.

**Conclusion:**

This is the first reported case of dysphonia accompanying severe dysphagia following left pneumonectomy. While postpneumonectomy syndrome is rare, a high degree of clinical suspicion is recommended when treating patients with history of pneumonectomy.

## 1. Introduction

Postpneumonectomy syndrome is a rare late-stage complication of pneumonectomy. It is characterized by a shift of the mediastinum into the postpneumonectomy space, with compression of the distal trachea and left main bronchus between the aorta and the pulmonary artery. Common symptoms include dyspnea, recurrent infections, and wheezing [1, 2]. However, dysphagia is infrequently mentioned. The physiologic consequences of pneumonectomy lead to midesophageal tortuosity and obstruction from the shift of the mediastinum, trapping of the esophagus between the heart and aorta causing esophageal dysmotility, and dilation or compression, ultimately leading to severe dysphagia.

We report a case of a patient who developed severe dysphagia and dysphonia 3 years after a left pneumonectomy. We present our diagnostic approach in order to make recommendations for similar presentations of this rare complication.

## 2. Case Presentation

**Figure 1:**
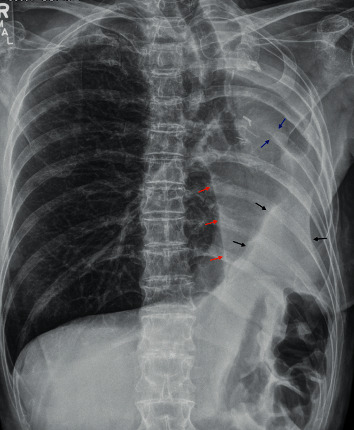
Anteroposterior chest radiograph demonstrates pneumonectomy surgical clips, suture, and left thoracostomy (blue arrows) with many secondary signs of left hemithorax volume loss including leftward tracheal shift, leftward shifting of the azygoesophageal recess (red arrows), upward tenting of the left hemidiaphragm (black arrows), and ipsilateral intercostal space narrowing.

**Figure 2:**
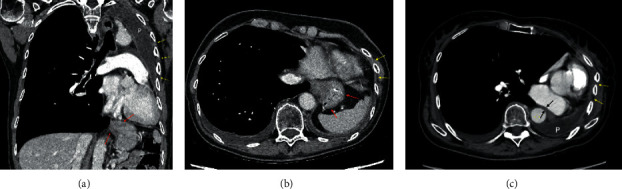
Coronal (a) and axial (b, c) reformats on contrast-enhanced chest CT more saliently depict the leftward cardiomediastinal shift and ipsilateral intercostal space narrowing (yellow arrows). Expected postsurgical fluid filling the pneumectomy space is present only along the superoposterior left hemithorax (labeled P). A small hiatal hernia is present (red arrows), which may be partly due to the tortuous course of the esophagus in the left hemithorax and consequent upward traction on the stomach. There may be some extrinsic compression on the esophagus (black arrows) between the left atrium and descending thoracic aorta, although an obstruction or poor distensibility was suggested on esophagram.

A 66-year-old female developed severe dysphagia, dyspnea, and dysphonia over the course of 18 months, 3 years following left pneumonectomy, radiotherapy, and immunotherapy for a stage IIIA adenocarcinoma of the lung. She described dysphagia as a sensation of solid foods and pills getting stuck in her throat and at the sternal notch and reported tasting her pills as they were dissolving. A repeated CT scan of the chest showed a leftward shift of mediastinal structures into postpneumonectomy space, but with no clear evidence of extrinsic tracheobronchial compression (Figures 1 and 2).

**Figure 3:**
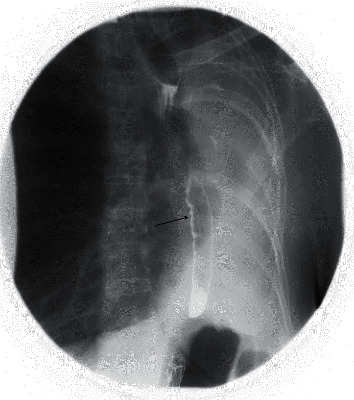
Anteroposterior spot image on double contrast esophagram demonstrates a leftward deviated esophagus that was appropriately distensible and without fixed narrowing. Expected primary stripping waves were present, and there was no delay in transit of oral contrast through the esophagus. Nonspecific tertiary waves were noted in the distal esophagus (black arrow) but is commonly seen in older patients with presbyesophagus.

Flexible fiberoptic laryngoscopy and stroboscopy demonstrated paresis of the left vocal fold with preserved complete glottic closure upon phonation. Functional endoscopic evaluation of swallowing showed a functional oropharyngeal swallow without signs of aspiration or penetration. Transnasal esophagoscopy demonstrated tortuous esophagus without stenosis. The cervical esophagus was noted to make a near 90 degree angle turn. A subsequent esophagram showed that the esophagus traverses to the left of midline, related to prior left-sided pneumonectomy, but with no signs of fixed narrowing (Figure 3). Modified barium swallow revealed again deflection of the esophagus to the left of the midline and sequestration of dense solids and pills in this tortuous portion of the cervical esophagus. This patient was treated with voice therapy, swallow therapy, and an exclusion diet. She elected not to move forward with surgery as mediastinal repositioning is a major undertaking with significant risks and morbidities. During follow-up visits, the patient has reported marked improvement in her swallow and voice function.

## 3. Discussion

Postpneumonectomy syndrome is a rare entity that occurs secondary to excessive mediastinal shift into the evacuated cavity. It has an incidence of 0.16% in patients following pneumonectomy [9]. While most commonly associated with dyspnea, it can also cause esophageal dysmotility and dysphagia [1, 2]. No other case of dysphonia accompanying post-pneumonectomy syndrome has been reported. This patient was noted to have decreased left vocal fold mobility. The authors surmise that this resulted from stretching of the left recurrent laryngeal nerve as it deviated with the esophagus far to the left of midline.

The mechanism of functional impairment in postpneumonectomy is clearer in patients who develop the condition following right pneumonectomy than left. In such cases, the counterclockwise rotation of the mediastinal structures causes compression of the left main bronchus between the aorta and pulmonary artery. Following left pneumonectomy, on the other hand, the clockwise rotation of mediastinal structures may result in compression of trachea and contralateral bronchial structures between the aorta and vertebral column [3]. A right aortic arch is a precondition for left postpneumonectomy syndrome, more rarely occurring in patients with normal aortic arches. The bronchus may appear open in the CT scans of patients presenting with left postpneumonectomy syndrome, in contrast to those with right postpneumonectomy syndrome [2]. Therefore, diagnosis of left postpneumonectomy syndrome may be made by exclusion if no other symptoms are reported.

If the patient presents with dysphagia, radiographic imaging including esophagram is the primary means of diagnosis. If left untreated, postpneumonectomy syndrome may result in tracheobronchomalacia [1, 2]. The patient presented here opted to forego treatment. However, treatment in other cases has included endobronchial stenting, phrenectomy, mediastinal repositioning, and pericardial fixation. Our patient primarily complained of progressive dysphagia and dysphonia. The previously reported treatments address dyspnea rather than dysphagia or dysphonia. While mediastinal repositioning may have been a viable option, the patient was not interested in having a major surgery requiring a thoracotomy.

In a comprehensive literature search of PubMed from 1980 to 2020 using the terms postpneumonectomy, dysphagia, and/or esophagus, 6 other documented cases of severe dysphagia associated with postpneumonectomy syndrome were identified. Dysphagia is most common after right pneumonectomy and is more common in men than women. Other classic symptoms include stridor, dry cough, difficulty managing secretions, recurrent pneumonia, and progressive dyspnea. While it is accompanied by dyspnea in all reported cases, no other cases of dysphonia were noted [3–8]. Reported symptoms tend to worsen over time. Esophageal dysmotility has also been identified in postpneumonectomy patients without a complaint of dysphagia [1]. These patients may present with severe dysphagia anywhere from 1 month to 23 years following pneumonectomy, as onset of symptoms is typically gradual. Treatment typically involves mediastinal repositioning via insertion of saline prosthesis [3–5], or an exclusion diet if surgery is deemed too risky [6, 7].

Although postpneumonectomy syndrome is a rare cause of dysphagia, it must be considered in the patient with a significant cardiothoracic or pulmonary history. Clinical examination may point to cardiogenic origin of dyspnea and heartburn, especially if no tracheobronchial compression is evident in radiographic imaging [2].

## 4. Conclusion

Our case represents the first reported case of dysphonia accompanying severe dysphagia following a left pneumonectomy. Diagnosis of left postpneumonectomy syndrome can be complicated by the fact that the bronchus may appear widely patent on chest CT scan. Furthermore, the esophagram may not reveal any clear evidence of extrinsic compression, despite the suggestion of compromised motility during swallowing. The key to accurate diagnosis is esophageal motility studies including modified barium swallow and manometry.

## Data Availability

Data will be made available upon request to the corresponding author.

## Additional Points

*Summary*. (i) Postpneumonectomy syndrome is extremely rare, occurring in less than 0.016% of patients. (ii) It occurs as a result of clockwise (left) or counterclockwise (right) rotation of mediastinal structures into the postpneumonectomy space, causing tracheobronchial compression. (iii) Postpneumonectomy syndrome occurs most commonly following a right pneumonectomy and is typically associated with dyspnea. (iv) This is the first reported case of dysphonia accompanying severe dysphagia following left pneumonectomy. (v) Patients presenting with heartburn and dysphagia with a history of pneumonectomy should be treated with a high degree of clinical suspicion.
